# Safety and effectiveness of the first balloon-in-basket pulsed field ablation system for the treatment of atrial fibrillation: VOLT CE Mark Study 6-month results

**DOI:** 10.1093/europace/euaf072

**Published:** 2025-03-31

**Authors:** Roland R Tilz, Gian Battista Chierchia, Melanie Gunawardene, Prashanthan Sanders, Haris Haqqani, Jonathan Kalman, Stewart Healy, Helmut Pürerfellner, Petr Neuzil, Joaquín Osca Asensi, Peter Loh, Vivek Y Reddy, Sébastien Knecht, Emily Jesser, Nick Dirckx, Amber Miller, Daniel Walker, Dhanunjaya Lakkireddy

**Affiliations:** Department of Rhythmology, University Heart Center, University Hospital Schleswig-Holstein, Lübeck, Germany; German Center for Cardiovascular Research (DZHK), Partner Site Hamburg/Kiel/Lübeck, Lübeck, Germany; Heart Rhythm Management Center, UZ Brussels -Vrij Universiteit Brussel, Brussels, Belgium; Department of Cardiology and Intensive Care Medicine, Asklepios Hospital St. Georg, Hamburg, Germany; Centre for Heart Rhythm Disorders, University of Adelaide and Royal Adelaide Hospital, Port Rd, Adelaide SA 5000, Australia; Department of Cardiology, The Prince Charles Hospital, 627 Rode Rd, Chermside QLD 4032, Australia; Department of Cardiology, University of Melbourne, Royal Melbourne Hospital—City Campus, 300 Grattan Street, Parkville, Victoria 3050, Australia; Department of Cardiology, Victorian Heart Hospital, Monash Health, 246 Clayton Road, Clayton, Victoria 3168, Australia; Department of Internal Medicine II with Cardiology, Angiology, and Intensive Care Medicine, Ordensklinikum Linz Elisabethinen, Linz, Austria; Cardiology Department, Nemocnice Na Homolce, Roentgenova 37, 150 00 Praha 5, Czechia; Division of Cardiac Arrhythmias and Electrophysiology, Cardiology Department, Hospital Universitari i Politecnic La Fe, Avinguda de Fernando Abril Martorell, 106, Quatre Carreres, 46026 València, Valencia, Spain; Department of Cardiology, UMC Utrecht, Heidelberglaan 100, Utrecht, 3584 CX, Netherlands; Cardiology Department, Nemocnice Na Homolce, Roentgenova 37, 150 00 Praha 5, Czechia; Mount Sinai Fuster Heart Hospital, One Gustave L. Levy Medical Place, New York, New York, 10029, USA; Department of Cardiology, AZ Sint Jan, Ruddershove 10, 8000 Brugge, Belgium; Electrophysiology, Abbott, Minneapolis, MN, USA; Electrophysiology, Abbott, Minneapolis, MN, USA; Electrophysiology, Abbott, Minneapolis, MN, USA; Electrophysiology, Abbott, Minneapolis, MN, USA; Kansas City Heart Rhythm Institute and Research Foundation, 5100 W 100th St, Suite-200, Overland Park, KS 66211, USA

**Keywords:** Pulmonary vein isolation, Catheter ablation, Pulsed field ablation, Balloon-in-basket, Atrial fibrillation, Balloon, Basket, Single-shot

## Abstract

**Aims:**

Pulsed field ablation (PFA) is a growing ablation modality for pulmonary vein isolation (PVI) in atrial fibrillation (AF) patients. This study assesses the 6-month safety and effectiveness of a novel balloon-in-basket, mapping-integrated PFA system, with a purpose-built form factor for PVI.

**Methods and results:**

The VOLT CE Mark Study is a prospective, multi-center, pre-market study. A total of 150 patients with drug-refractory paroxysmal (PAF) or persistent AF (PersAF) were enrolled between 8 November 2023 and 14 March 2024, of which 146 patients (age 64.1 ± 10.0 years, 63.0% male, 70.5% PAF) underwent PVI with the balloon-in-basket PFA catheter and system featuring integrated electroanatomic mapping with contact-sensing. Study endpoints were the rate of primary serious adverse events within 7 days as well as acute procedural effectiveness and 6-month freedom from recurrence. Acute effectiveness was achieved in 99.1% (573/578) of treated PVs (98.6% of patients, 144/146) with 17.6 ± 5.7 PFA applications/patient. Procedure, fluoroscopy, LA dwell, and transpired ablation times were 100.4 ± 33.0, 17.3 ± 12.1, 39.4 ± 20.6, and 31.4 ± 16.8 min, respectively. There were 4 (2.7%; 4/146) primary serious adverse events. The rate of freedom from documented atrial arrhythmias was 88.2% in PAF patients and 76.7% in PersAF patients (freedom from symptomatic recurrence was documented in 90.2% of PAF patients and 74.4% of PersAF patients) through 6-months post-index procedure.

**Conclusion:**

The VOLT CE Mark Study primary results demonstrate the safety and effectiveness of the novel balloon-in-basket PFA system to perform PVI in PAF and PersAF.

## Graphical Abstract

**Graphical Abstract:**
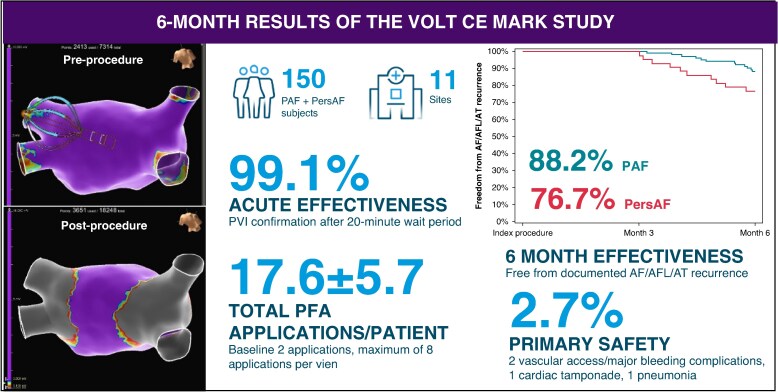

**What’s new?**

- This is the first study worldwide to report on the safety and acute and chronic effectiveness of pulmonary vein isolation using a novel balloon-in-basket pulsed field ablation catheter.
- The novel ablation system, with a purpose-built form factor for PVI, was found to be safe, with a 2.7% primary endpoint adverse event rate.
- Acute and chronic effectiveness were high in paroxysmal and persistent atrial fibrillation patients. Acute isolation was achieved in 573 of 578 [veins (99.1%) treated pulmonary]. The rate of freedom from documented atrial arrhythmias was 88.2% in PAF patients and 76.7% in PersAF patients at 6 months.

## Introduction

Catheter ablation with pulmonary vein isolation (PVI) has become an effective treatment of symptomatic, drug-refractory atrial fibrillation (AF) with traditional therapies including radiofrequency (RF) and cryotherapy modalities.^1^ Single-shot catheter designs for PVI with thermal energy sources have been shown to deliver reproducible results and offer a short learning curve for operators.^2,3^

However, despite their clinical success, thermal ablation modalities, such as RF and cryoablation, are associated with collateral damage complications, including potential injury to the oesophagus or phrenic nerve, and pulmonary vein (PV) stenosis.^4,5^ In response to these challenges, pulsed field ablation (PFA) has emerged as an innovative alternative for catheter ablation.^6^ Unlike thermal methods, PFA utilizes high-voltage electrical fields to induce irreversible electroporation of cardiomyocytes. Differing tissue sensitivity to these fields offers the potential of preferential-myocardial ablation and safer lesion formation. The favourable safety profile of PFA and observed ability to minimize collateral damage has been reported before.^7–9^ Nevertheless, opportunities remain to enhance the effectiveness of circumferential lesion delivery and durability, safety, and workflows, and to minimize the necessity of repeat ablations after initial treatment with PFA systems.^10^

A recent advancement in PFA technology is the development of the novel balloon-in-basket catheter with a purpose-built form factor for optimal PVI and designed with contact-sensing capabilities fully integrated into an electroanatomic three-dimensional (3D) mapping system. This PFA system has been evaluated in an early feasibility sub-study of 32 patients, demonstrating acute safety and effectiveness in the treatment of symptomatic, drug-refractory AF.^11^

The VOLT CE Mark Study was performed to evaluate the 6-month safety and effectiveness of the novel balloon-in-basket PFA system for treatment of paroxysmal (PAF) and persistent atrial fibrillation (PersAF).

## Methods

### Trial design

The VOLT CE Mark Study (NCT06106594) was a pre-market, prospective, non-randomized, multi-center, first-in-human clinical investigation designed to investigate the safety and acute and long-term effectiveness of the Volt™ PFA system for the treatment of symptomatic, recurrent and drug-refractory PAF and PersAF between November 2023 and March 2024. This included a feasibility sub-study that was previously reported.^11^ We here report the safety and acute and 6-month effectiveness of the novel Volt™ PFA system.

This study was sponsored by Abbott with oversight by a steering committee, publication committee, clinical events committee, and data safety monitoring board. Written informed consent was obtained from all patients. The study was conducted in accordance with the provisions of the Declaration of Helsinki and its amendments. At each center, local ethics committees approved the study.

### Study participants

Patients were ≥18 years of age, willing and able to consent to the procedure and follow-up and planned to undergo an ablation procedure for symptomatic drug-refractory PAF (recurrent self-terminating AF within 7 days) or PersAF (continuous AF sustained beyond 7 days and <1 year). To be eligible, patients had to have electrocardiographically documented AF episodes within 12 months prior to informed consent and a physician’s note indicating their type of AF. Key exclusion criteria were left atrial (LA) diameter >55 mm, left ventricular ejection fraction <35%, New York Heart Association (NYHA) functional class III or IV, body mass index >40 kg/m^2^, previous ablation therapy in the LA and the presence of any implantable cardiac devices.

### Ablation procedure and mapping

Ablation methods with the Volt™ PFA system have been previously described.^11^ Investigators were recommended to maintain their patients on an uninterrupted oral anticoagulation regimen. Procedures were performed under general anaesthesia or conscious/deep sedation as per site standard of care. Left atrial appendage thrombus was ruled out via CT or trans-esophageal echocardiography (TEE) within a day of the procedure start. Prior to ablation, the phrenic nerve was paced from the superior vena cava (SVC) to test for a baseline phrenic nerve function for comparison to repeat pacing at the end of the procedure. An activated clotting time of >300 s was recommended for the duration of LA access. Transseptal LA access was obtained using fluoroscopy, intracardiac echocardiography and/or TEE guidance according to operator preference. A multipolar mapping catheter was used to create a pre-ablation three-dimensional geometry with voltage mapping of the LA. The mapping catheter was then removed, and the transseptal sheath was exchanged with the 13F sheath, through which the Volt™ PFA catheter could be introduced after preparation. Use of a maximum diameter 0.035 inch guidewire was deployed via the through-lumen in the Volt catheter to help engage veins and provide stability for the device in the vein antrum/ostium. PFA therapy was delivered at either 1800V (nominal) or at 1400V (low) with a minimum of two applications (and device rotation between applications) with a nominal waveform and three applications (with device rotation between each application) with a low waveform. When treating the right-sided PVs, pacing from the Volt™ PFA catheter was performed to test for phrenic nerve proximity. In the event of phrenic nerve capture, the low-voltage waveform therapy was employed. Any subsequent repositioning of the device required additional pacing to check for phrenic capture. After PVI, a 20-min waiting period was required. For any vein, a maximum of eight therapy applications was allowed. Following ablation, a post-ablation voltage map was generated and phrenic nerve function via pacing in the SVC was confirmed. Echocardiography was performed to rule out pericardial effusion. Post-procedural care and management were conducted according to standard practices at the local institutions.

### Follow-up

Scheduled follow-up occurred at 7, 30 days, 3 and 6 months. Follow-up is ongoing and will complete at the 12-month visit for all patients. A 90-day blanking period was observed after the index ablation procedure. Medication adjustments, cardioversions, and one repeat ablation procedure utilising the Volt™ PFA system were allowed during the blanking period without being considered a primary effectiveness endpoint failure. LA voltage mapping was required for any clinically indicated LA repeat ablation procedure. At 30 days, patients were reminded to discontinue antiarrhythmic drugs (AADs) unless clinically justified. Subjects must be off all Class I and III AADs prescribed for AF/AFL/AT after the blanking period, unless clinically justified and deemed necessary by the investigator.

A 12-lead electrocardiogram (ECG) was performed at the 3-month visit. At least one trans-telephonic transmission was required to be collected in every 14-day period between the 3- and 6-month visits. At 6 months, a 24-h Holter monitor was administered.

### Endpoints

The primary safety endpoint was defined as the rate of patients experiencing a pre-defined device and/or procedure-related serious adverse event with onset within 7-days of any ablation procedure (index or repeat procedure) that used the Volt™ PFA system. The pre-defined primary safety events include the following: atrio-esophageal fistula, cardiac tamponade/perforation, death, heart block, myocardial infarction, pericarditis (only considered a primary safety endpoint event if it results in a pericardial effusion that leads to hemodynamic compromise, requires pericardiocentesis, prolongs or requires hospitalisation more than 48 h for reasons other than observational purposes, or persists for more than 30 days following the ablation procedure), phrenic nerve injury resulting in permanent diaphragmatic paralysis, pulmonary oedema, PV stenosis, stroke/cerebrovascular accident, thromboembolism, transient ischemic attack, vagal nerve injury/gastroparesis, major vascular access complications (defined as one that requires intervention, such as surgical repair, vascular catheter intervention or thrombin injection, or prolongs the hospital stay or requires hospital admission >24 h for reasons other than for observational purposes)/major bleeding events [considered a primary endpoint event if it requires and/or is treated with transfusion (≥2 units) or results in a 20% or greater fall in hematocrit], and device and/or procedure-related cardiovascular and/or pulmonary adverse event that prolongs hospitalisation for more than 48 h (excluding hospitalisation solely for arrhythmia recurrence or non-urgent cardioversion). Other adverse events considered reportable were events that were cardiovascular related or assessed by the investigator as being related to (including those possibly related to) either the ablation catheter or the ablation procedure and all serious adverse events, regardless of relatedness to the procedure and/or the ablation catheter.

Acute procedural success was defined as the rate of isolated PVs treated with the Volt™ PFA system at the end of the index ablation procedure. Acute procedural failure for each PV was defined as any of the following: (i) an inability to isolate a PV at the end of the index ablation procedure (with a maximum allowance of eight PFA applications) assessed via confirmation of electrical isolation in each ablated PV after a minimum waiting period of 20 min via entrance block at a minimum; (ii) any use of a non-study ablation device for PVI. First-pass isolation was defined as confirmation of entrance block in all treated PVs following the initial minimum 20-min waiting period without the need for additional ablation after the start of the 20-min waiting period.

The primary effectiveness endpoint was defined as a composite of acute and chronic success. This includes freedom from documented (symptomatic or asymptomatic) atrial arrhythmia [AF, atrial flutter (AFL), or atrial tachycardia (AT)], which was defined as AF/AFL/AT episodes >30 s in duration documented by protocol-specified 12-lead ECG, TTM, or Holter monitoring after the index ablation procedure through 6 months of follow-up (after 90-day blanking period through 181 days post-index ablation procedure). Patients were considered primary effectiveness endpoint failures in the following situations: (i) acute procedural failure as defined previously; (ii) any use of a non-study ablation device for PVI or to deliver ablation lesions in the LA during the index procedure or during the first repeat procedure; (iii) documented AF, AFL, or AT recurrence (>30 s episode), excluding cavotricuspid-dependent AFL that has been confirmed in an electrophysiology study (e.g. via entrainment maneuvers), at any time after the blanking period (>90 days after the index procedure) as assessed by protocol-specified 12-lead ECG, TTM, and Holter monitoring; (iv) a repeat procedure for the treatment of AF, non-CTI-dependent AFL, or AT after the blanking period or a second repeat AF ablation procedure at any time after the index ablation procedure; (v) any use of a new class I or III AAD for AF after the blanking period; (vi) any use of a class I or III AAD for AF at a dose higher than the historical maximum dose for the patient after the blanking period; (vii) electrical or pharmacological cardioversion for the treatment of AF/AFL/AT after the blanking period (excluding CTI-dependent AFL confirmed by entrainment maneuvers); (viii) surgical treatment of AF/AFL/AT post-index procedure. The situations that were not considered effectiveness endpoint failures included: (i) AF/AFL/AT recurrence during the 90-day blanking period (≤90 days post-index procedure); (ii) one repeat procedure for ablation of AF recurrence during the 90-day blanking period (performed 31–80 days post-ablation) in which the Volt™ PFA catheter was used for PVI; (iii) use of previously failed Class I or III AADs taken at doses that do not exceed the maximum failed dose prior to the index procedure after the 90-day blanking period. Symptomatic effectiveness is defined similarly to the primary effectiveness endpoint with the exception that documented recurrence without documented evidence of symptoms after the 90-day blanking period was not considered a failure.

### Statistical methods

Continuous variables are reported as mean ± SD, median, and IQR, irrespective of the normality of their distribution. Categorical variables are expressed as number (percent). Primary effectiveness at 6-Months was estimated using Kaplan-Meier analysis with Greenwood standard error. Analyses were performed by using SAS version 9.4 (SAS Institute, Inc.).

## Results

### Baseline characteristics

**Figure 1:**
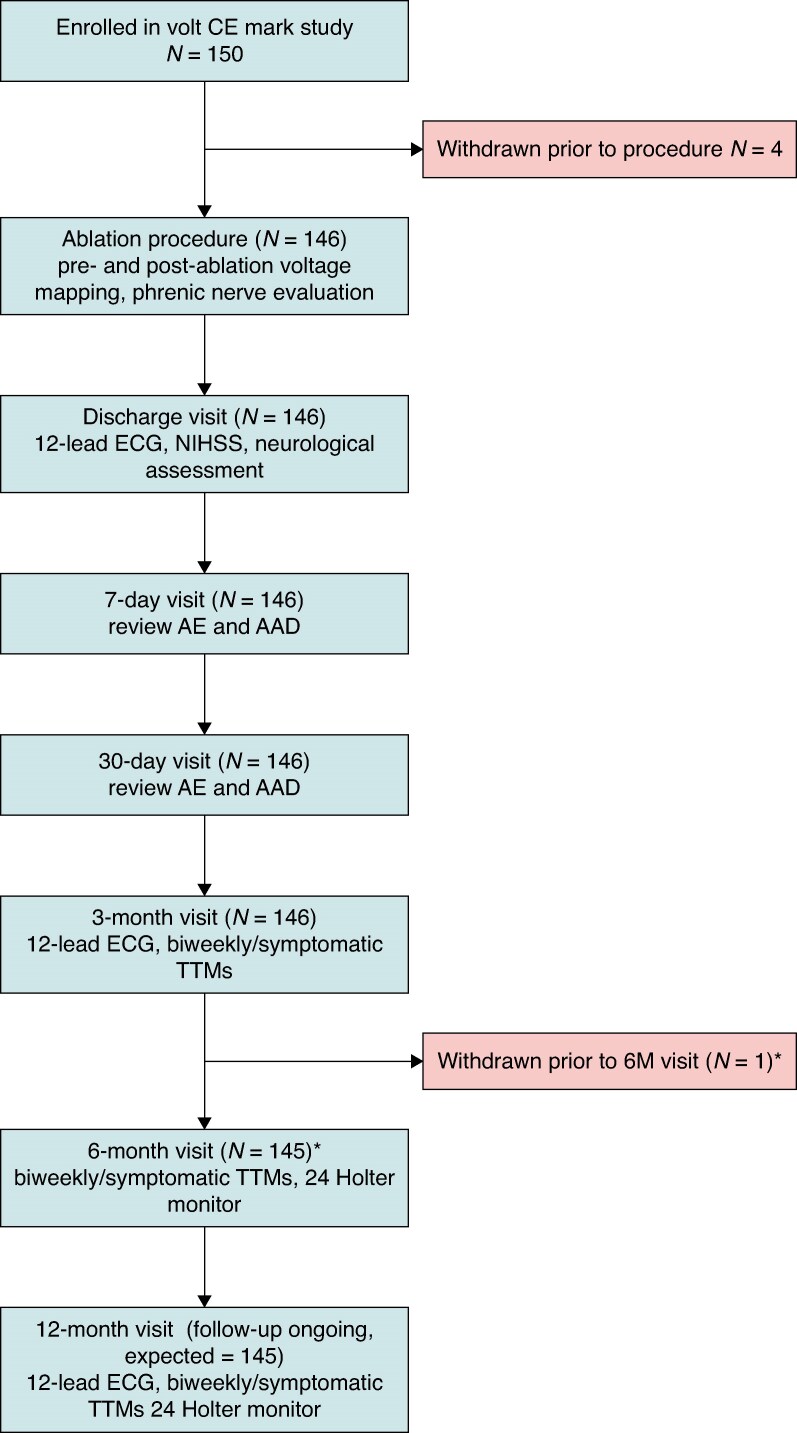
Subject disposition and follow-up events. Follow-up though 6 months is complete for the study. 12-month follow-up is ongoing. *One patient withdrew from the study prior to completing their 6-month visit; however, this subject experienced an endpoint event before withdrawing and therefore is included in the primary effectiveness endpoint. Four patients were enrolled but withdrawn prior to the insertion of the investigational PFA system due to mechanical bed malfunction, failure to meet inclusion/exclusion criteria, and PFA generator malfunction.

A total of 150 patients were enrolled in the VOLT CE Mark Study at 11 European and Australian sites between 8 November 2023 and 14 March 2024. Of the 150 enrolled, 146 patients (mean age 64.1 ± 10.0 years, 63.0% male, 70.5% PAF) underwent ablation with the investigational system. Patient enrolment and follow-up are presented in *Figure 1*. *Table 1* summarizes the baseline characteristics of the patients treated. A total of 70.5% (103/146) patients enrolled had PAF. In the PAF population (mean age 63.3 ± 10.7 years, 58.3% male), the median CHA_2_DS_2_-VASc score was 2.0, IQR (1.0, 3.0) and hypertension was the most frequent comorbidity (53.4%, 55/103). In the PersAF population (29.5%; 43/146) (mean age 65.9 ± 8.0, 74.4% male), the median CHA_2_DS_2_-VASc score was 2.0, IQR (1.0, 3.0), with hypertension as the most common comorbidity, as well (60.5%, 26/43). All but two patients received at least one class I-IV AADs to treat AF.

**Table 1:** Patient characteristics

	PAF (*n* = 103)	PersAF (*n* = 43)	All (*n* = 146)
Age (years)			
Mean ± SD (*n*)	63.3 ± 10.7 (103)	65.9 ± 8.0 (43)	64.1 ± 10.0 (146)
Median (Q1, Q3)	64.0 (57.0, 71.0)	65.0 (62.0, 70.0)	64.5 (58.0, 71.0)
BMI (kg/m^2^)			
Mean ± SD (*n*)	28.6 ± 4.7 (103)	30.4 ± 4.8 (43)	29.1 ± 4.8 (146)
Median (Q1, Q3)	27.8 (25.6, 30.8)	30.1 (27.1, 34.3)	28.4 (26.2, 31.7)
Gender, % (*n*/*N*)			
Female	41.7% (43/103)	25.6% (11/43)	37.0% (54/146)
Male	58.3% (60/103)	74.4% (32/43)	63.0% (92/146)
Race, % (*n*/*N*)			
American Indian or Alaska Native	0.0% (0/103)	0.0% (0/43)	0.0% (0/146)
Asian	0.0% (0/103)	0.0% (0/43)	0.0% (0/146)
Black or African American	0.0% (0/103)	0.0% (0/43)	0.0% (0/146)
Native Hawaiian or other Pacific Islander	0.0% (0/103)	0.0% (0/43)	0.0% (0/146)
White	34.0% (35/103)	30.2% (13/43)	32.9% (48/146)
Patient refused to provide	66.0% (68/103)	69.8% (30/43)	67.1% (98/146)
Ethnicity, % (*n*/*N*)			
Hispanic or Latino	0.0% (0/103)	0.0% (0/43)	0.0% (0/146)
Not Hispanic or Latino	33.0% (34/103)	30.2% (13/43)	32.2% (47/146)
Patient refused to provide	67.0% (69/103)	69.8% (30/43)	67.8% (99/146)
NYHA classification, % (*n*/*N*)			
I	30.1% (31/103)	32.6% (14/43)	30.8% (45/146)
II	4.9% (5/103)	20.9% (9/43)	9.6% (14/146)
III	0.0% (0/103)	0.0% (0/43)	0.0% (0/146)
IV	0.0% (0/103)	0.0% (0/43)	0.0% (0/146)
No heart failure, % (*n*)	65.0% (67/103)	46.5% (20/43)	59.6% (87/146)
CHA_2_DS_2_ -Vasc score			
Mean ± SD (*n*)	1.9 ± 1.4 (103)	2.1 ± 1.7 (43)	1.9 ± 1.5 (146)
Median (Q1, Q3)	2.0 (1.0, 3.0)	2.0 (1.0, 3.0)	2.0 (1.0, 3.0)
LVEF (%)			
Mean ± SD (*n*)	60.2 ± 6.4 (101)	53.3 ± 8.6 (41)	58.2 ± 7.7 (142)
Median (Q1, Q3)	60.0 (55.0, 65.0)	55.0 (46.0, 60.0)	60.0 (55.0, 65.0)
LA Diameter (mm)			
Mean ± SD (*n*)	39.9 ± 6.7 (101)	42.3 ± 8.1 (41)	40.6 ± 7.2 (142)
Median (Q1, Q3)	40.0 (36.0, 45.0)	43.0 (39.0, 45.0)	41.0 (38.0, 45.0)
Medical history			
Hypertension	53.4% (55/103)	60.5% (26/43)	55.5% (81/146)
Patient has taken at least one Class I/III AAD, % (*n*/*N*)	75.7% (78/103)	65.1% (28/43)	72.6% (106/146)
Patient has taken at least one Class II/IV AAD, % (*n*/*N*)	71.8% (74/103)	90.7% (39/43)	77.4% (113/146)
Anticoagulation, % (*n*/*N*)	91.3% (94/103)	100.0% (43/43)	93.8% (137/146)
Ongoing at baseline, % (*n*/*N*)	82.5% (85/103)	83.7% (36/43)	82.9% (121/146)

Baseline characteristics and demographics of patients who underwent ablation with the Volt™ PFA catheter.

### Procedural characteristics

The average procedure time, including the protocol-mandated 20-min waiting period, pre- and post-procedure phrenic nerve stimulation, and voltage mapping, was 100.4 ± 33.0 min with an average 39.4 ± 20.6 min Volt™ PFA catheter LA dwell time. The average total fluoroscopy time was 17.3 ± 12.1 min. The transpired ablation time (from the first PFA application to the last application) was 31.4 ± 16.8 min. Procedural characteristics are summarized in *Table 2*. There was one outlier in procedural times due to difficulty accessing one right inferior PV in one patient.

**Table 2:** Procedural characteristics

	PAF (*N* = 103)	PersAF (*N* = 43)	All (*N* = 146)
Procedure time (min)			
Mean ± SD (*n*)	99.6 ± 31.3 (103)	102.3 ± 37.1 (43)	100.4 ± 33.0 (146)
Median (Q1, Q3)	95.0 (76.0, 116.0)	93.0 (79.0, 131.0)	94.5 (77.0, 117.0)
Fluoroscopy time (min)			
Mean ± SD (*n*)	16.2 ± 9.9 (102)	20.0 ± 16.1 (43)	17.3 ± 12.1 (145)
Median (Q1, Q3)	13.0 (9.0, 20.0)	14.0 (8.6, 26.0)	13.7 (9.0, 20.1)
LA Dwell time (min)			
Mean ± SD (*n*)	39.1 ± 19.3 (103)	40.1 ± 23.5 (43)	39.4 ± 20.6 (146)
Median (Q1, Q3)	36.0 (27.0, 49.0)	34.0 (23.0, 45.0)	35.0 (26.0, 49.0)
Transpired ablation time (min)			
Mean ± SD (*n*)	31.3 ± 16.7 (103)	31.5 ± 17.1 (43)	31.4 ± 16.8 (146)
Median (Q1, Q3)	28.0 (22.0, 40.0)	27.0 (21.0, 37.0)	27.0 (21.0, 39.0)

LA, left atrial.

Total procedure time is defined as first venous puncture to the time either groin sheaths were pulled, or patient left the EP lab (whichever occurred first). LA dwell time is defined is defined as time Volt™ PFA catheter entered the LA to the time the Volt™ PFA catheter exited the LA. Transpired ablation time is defined as time of first PFA therapy application to last PFA therapy application.

**Figure 2:**
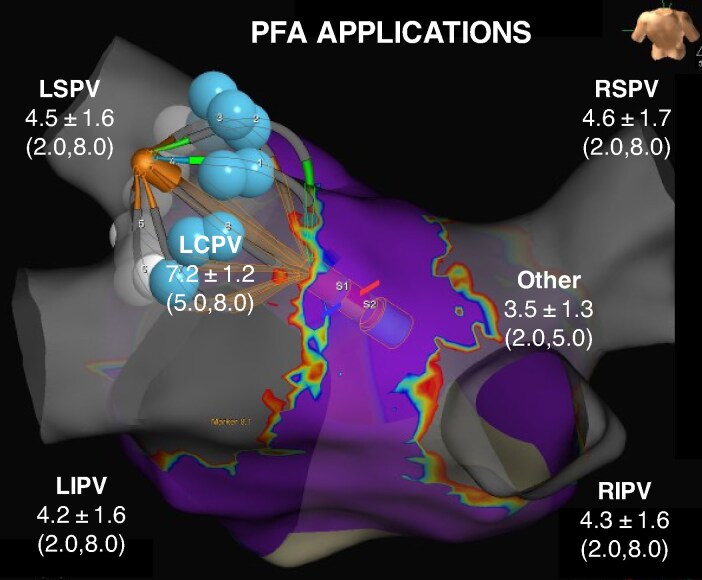
PFA therapy applications per PV location. Mean ± SD and (min, max) values for the number of therapy applications delivered in each PV location. LSPV denotes left superior pulmonary vein, LIPV left inferior pulmonary vein, LCPV left common pulmonary vein, RSPV right superior pulmonary vein, RIPV right inferior pulmonary vein, and Other denotes a PV not matching one of the preceding classifications (1 roof vein and 3 right middle veins).

An average of 17.6 ± 5.7 PFA therapy applications were delivered per patient (*Figure 2*). Phrenic nerve function was confirmed prior to PFA therapy delivery in all procedures and was confirmed at the end of the procedure in all but one procedure due to pericardial effusion during procedure.

### Safety

A primary safety endpoint event was experienced in 2.7% (4/146) of patients (2 PAF and 2 PersAF patients) (*Table 3*). There were 2 PAF patients with major vascular access complication. There was 1 PersAF patient with cardiac tamponade/perforation and 1 PersAF patient presented with pneumonia 5 days post-procedure requiring hospitalisation. Primary safety event details are further discussed in the Supplementary material. There were no deaths, phrenic nerve injury, or clinically significant coronary artery vasospasm or haemolysis through 6 months of follow-up.

**Table 3:** Primary safety endpoint events

	Primary safety (*N* = 146)	
Endpoint criteria	% patients	# of events
Atrio-esophageal fistula	0.0% (0/146)	0
Cardiac tamponade/perforation	0.7% (1/146)	1
Death	0.0% (0/146)	0
Heart block (AV block)	0.0% (0/146)	0
Myocardial infarction	0.0% (0/146)	0
Pericarditis	0.0% (0/146)	0
Phrenic nerve injury resulting in diaphragmatic paralysis	0.0% (0/146)	0
Pulmonary oedema	0.0% (0/146)	0
Pulmonary vein stenosis	0.0% (0/146)	0
Stroke/cerebrovascular accident	0.0% (0/146)	0
Thromboembolism	0.0% (0/146)	0
Transient ischemic attack	0.0% (0/146)	0
Vagal nerve injury/gastroparesis	0.0% (0/146)	0
Major vascular access complication/major bleeding	1.4% (2/146)	2
Device and/or procedure-related cardiovascular and/or pulmonary adverse event that prolongs hospitalisation for more than 48 h	0.7% (1/146)	1
Total	2.7% (4/146)	4

### Acute effectiveness

A total of 578 PVs were treated in the 146 patients during the index procedure. Isolation was achieved in 573 of 578 treated PVs (99.1%), and all PVs receiving PFA therapy were isolated in 144 of 146 patients (98.6%) at the end of the procedure (*Table 4*). The definition of acute success was not met in a total of five PVs from two PersAF patients. In one patient, initial PVI was achieved in all four PVs treated as assessed by entrance block following therapy delivery; however, a reconnection in the left superior PV, (LSPV) was identified after the 20-min waiting period that persisted after delivery of additional applications reached the maximum therapy dose of eight applications allowed per study protocol. PVI was persistent in the other three PVs after the 20-min waiting period. In the other patient, after PVI of all four veins, the presence of pericardial effusion was noted during the procedure, which transitioned to pericardial tamponade requiring intervention prior to completion of the 20-min wait period necessary for confirmation of acute effectiveness. There was one patient treated early in the operator’s study experience in which the right inferior PV could not be accessed due to acute angulation of the vein relative to the transseptal puncture site and significant rotated anatomy. This PV is excluded from acute effectiveness analyses as it did not receive PFA therapy. However, the patient met the definition of acute effectiveness success at the subject level, as all veins that received PFA therapy were isolated.

**Table 4:** Acute procedural success

	PAF (*N* = 103)	PersAF (*N* = 43)	All (*N* = 146)
	(*V* = 410)	(*V* = 168^a^)	(*V* = 578^a^)
**Acute procedural success**			
Per subject	100.0% (103/103)	95.3% (41/43)	98.6% (144/146)
Per vein	100.0% (410/410)	97.0% (163/168^a^)	99.1% (573/578^a^)
**First-pass isolation**			
Per subject	96.1% (99/103)	90.7% (39/43)	94.5% (138/146)
Per vein	98.3% (403/410)	95.8% (161/168^a^)	97.6% (564/578^a^)

^a^One RIPV could not be accessed during the procedure and did not receive PFA therapy and is therefore excluded from this analysis.

Overall, first-pass isolation was achieved in 97.6% (564/578) of treated veins and in 94.5% (138/146) of patients in which the PVs that received PFA energy were evaluated for isolation (*Table 4*). On a per vein level, first-pass isolation was achieved in 98.3% (403/410) of treated veins in the PAF population and in 95.8% (161/168) of treated veins in the PersAF populations. Of the 578 treated veins, 1.7% (10/578) of PVs reconnected during the initial 20-minute waiting period. Of the reconnected veins, the most common reconnection site was the LSPV (50.0%; 5/10) (see Supplementary material online, *Table S1*).

### Chronic effectiveness

**Figure 3:**
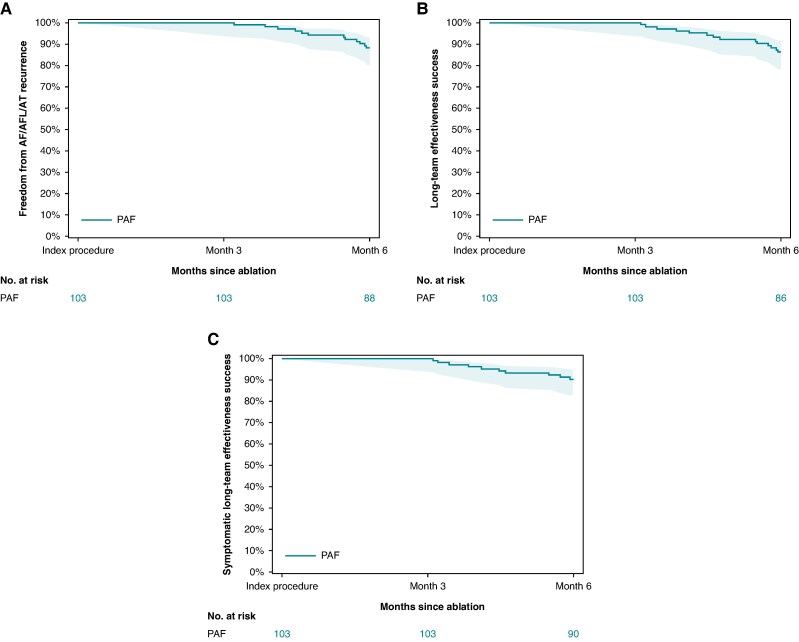
Effectiveness at 6 months in PAF patients. (*A*) Any AF/AFL/AT found on Holter, ECG, or TTM after the blanking period is considered an event. (*B*) Primary effectiveness endpoint of the study. (*C*) Symptomatic effectiveness.

**Figure 4:**
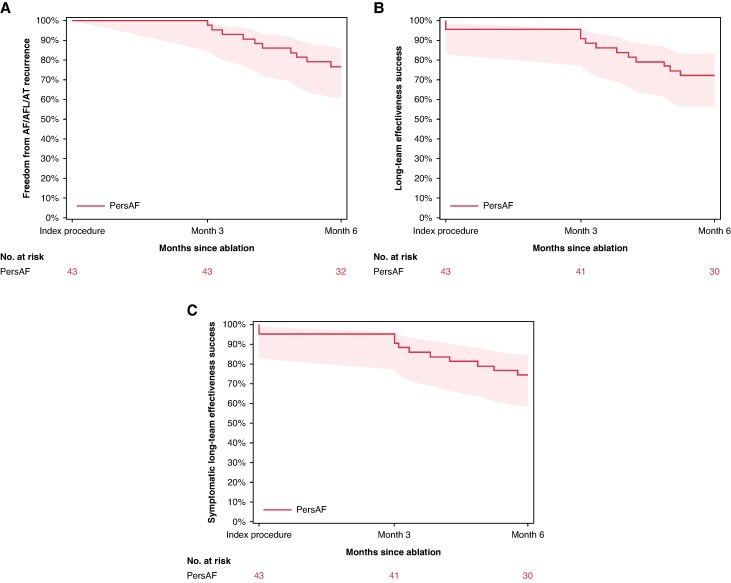
Effectiveness at 6 months in persistent atrial fibrillation patients. (*A*) Any AF/AFL/AT found on Holter, ECG, or TTM after the blanking period is considered an event. (*B*) Primary effectiveness endpoint of the study. (*C*) Symptomatic effectiveness.

Freedom from any AF/AFL/AT recurrence after the 90-day blanking period through 181 days post-index procedure was observed in 88.2% and 76.7% of the PAF and PersAF cohorts, respectively (*Figures 3A* and *4A* respectively). Freedom from the composite primary effectiveness endpoint at 6 months was 86.2% of PAF patients and 72.1% of PersAF patients (*Figures 3B* and *4B*, respectively). The reasons for effectiveness endpoint failure are summarized in Supplementary material online, *Table S2*. The rate of freedom from any symptomatic AF/AFL/AT recurrence was 90.2% in PAF patients and 74.4% in PersAF patients (*Figures 3C* and *4C*, respectively). Through 6-month follow-up, 27 patients (18.5%, 27/146) used Class I/III AADs after the blanking period (17 Class I; 10 Class III), with 2.7% (4/146) using amiodarone, specifically. In PAF and PersAF subjects who were completely off Class I/III AADs post-blanking period through the 6-month cut-off, 89.7% (78/87) and 84.4% (27/32) were free from AF/AFL/AT recurrence as determined by protocol-specified monitoring, respectively. Additional details of Class I/III AAD usage and effectiveness are found in the Supplementary material online.

### Repeat procedures and PV reconnections

**Figure 5:**
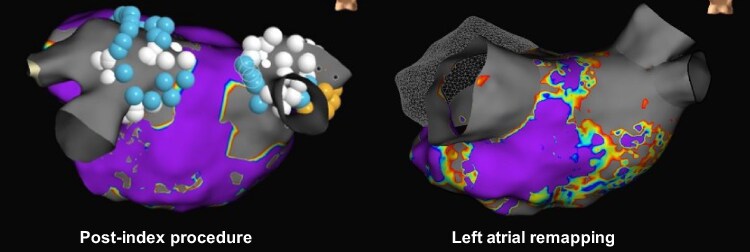
Remapping confirmed durable PVI in a repeat procedure. Remapping confirmed durable PVI 114 days post-index ablation procedure. Post-index (left) ablation voltage map and remapping of the left atrium prior to repeat ablation (right) are shown. This patient was treated for AF recurrence with anterior line, box isolation, and CTI line ablation. Arrhythmia was terminated.

Through 6 months of follow-up, there have been seven repeat procedures in seven patients (4/103; 3.9% of PAF patients and 3/43; 7.0% of PersAF patients) (see Supplementary material online, *Table S3*). Left atrial ablation with remapping was completed in six of the seven repeat procedures, with one repeat procedure requiring only right-sided ablation. Twenty-three total PVs were treated and isolated during the index procedures in patients who underwent repeat procedures and LA remapping. Therefore, the rate of durably isolated PVs in patients with clinically relevant recurrence requiring re-ablation at any time was 82.6% (19/23), and 93.3% (14/15) post-blanking period (*Figure 5*). Reconnections occurred in two patients: (i) One patient required a repeat ablation 63 days post-index procedure. Upon remapping, three of four (LSPV, LIPV, RSPV) veins were reconnected, and the patient was treated with repeat PVI using the Volt™ PFA catheter. All PVs were isolated at the end of the repeat procedure. (ii) The other patient required repeat ablation 142 days post-index procedure to treat typical AFL where the RSPV was found to be reconnected during remapping. This patient received repeat PVI and CTI line ablation with a commercially available catheter. (See Supplementary material online, *Table S4*).

## Discussion

The VOLT CE Mark Study was the first-in-human study to assess the safety and acute and chronic effectiveness of a novel balloon-in-basket PFA catheter, featuring electroanatomical mapping integration and tissue proximity-sensing, for PVI in patients with drug-refractory PAF and PersAF. The main findings are:

1. The novel balloon-in-basket PFA ablation system was found to be safe, with only 2.7% of patients experiencing primary safety endpoint events. There were no pulsed field energy-related adverse events.
2. Ablation with the system resulted in acceptable acute effectiveness and a high rate of freedom from any atrial arrhythmia recurrences in 88.2 and 76.7% of PAF and PersAF patients, respectively, at 6 months of post-ablation follow-up.

### Procedural safety

The incidence of primary safety endpoint events was remarkably low at 2.7%, with only two patients experiencing major vascular adverse events, one patient developing cardiac tamponade, and another presenting with pneumonia. Notably, there were no reports of death, or any pulsed field energy-related complications, such as phrenic nerve injury, esophageal fistula, clinically significant coronary artery vasospasm, or haemolysis.

Previous studies have highlighted the number of PFA applications as a critical factor influencing the risk of hemolysis.^12^ Another key determinant is the degree of tissue contact during energy delivery.^13^ Given the balloon-in-basket design of the VOLT™ PFA catheter, along with its integrated tissue contact monitoring and reduced electrode exposure to the blood pool, this risk appears to be significantly minimized. While earlier studies reported an acute kidney injury incidence of up to 3.2%, no cases of clinically significant hemolysis were observed in the present study.^12^ However, laboratory tests to evaluate non-clinically significant hemolysis were not systemically conducted as part of the study.

Furthermore, only one case of pericardial effusion, resulting in cardiac tamponade, was identified. In comparison to the penta-spline catheter design—which initially reported a cardiac tamponade incidence of up to 0.97%, primarily due to catheter manipulation and use of a stiff guidewire—the current findings suggest a reduced incidence, even among operators less familiar with the ablation system. Additionally, the tactile feedback from occlusion may enhance confidence in catheter positioning while simultaneously reducing the risk of pericardial effusion.^14^ Due to increased operator experience with balloon-based catheters used in cryoablations, the learning curve may be significantly shorter as compared to other designs.^15^ Whether these features will translate into tangible clinical benefits remains to be confirmed through clinical trials.

In contrast to the well-established cryoballoon, which can cause phrenic nerve palsy in up to 4.2% of cases reported in a registry^4^ and persistent phrenic nerve injury in 0.7% as shown in the ADVENT trial,^16^ no phrenic nerve injuries were reported in the VOLT CE Mark Study. Therefore, the VOLT CE Mark Study demonstrates the Volt™ PFA System has a favourable safety profile.

### Acute and chronic effectiveness

Acute effectiveness was demonstrated with 99.1% of PVs successfully isolated at the end of the waiting period. This result is comparable to other anatomical and single-shot ablation technologies, as a recent meta-analysis reported an acute procedural success rate of 99.9% for the penta-spline PFA catheter and 99.1% for cryoballoon ablation.^17^ It is notable that the high rate of acute effectiveness was achieved despite the VOLT CE Mark Study limited therapy delivery to a maximum of eight applications per vein, whereas early experiences with other PFA systems often involved more than eight applications per vein.^18,19^ There was one vein during the study that was not isolated after the maximum allowed therapy applications. Without this protocol limitation, there could have potentially been a higher acute isolation rate. Additionally, confirmation of PV isolation status after the 20-min waiting period was not possible in one patient due to the onset of pericardial tamponade, which required immediate intervention.

At 6 months, 86.2 and 72.1% of PAF and PersAF patients, respectively, remained free of the stringent composite primary effectiveness endpoint events, and 88.2 and 76.7% of PAF and PersAF patients were free from Holter-, ECG-, or TTM-documented AF/AFL/AT recurrence, respectively. Comparable studies of other PFA systems report effectiveness at 1 year, such as the ADVENT trial, in which the primary effectiveness endpoint was achieved by 73.3% of PAF patients in the PFA group.^16^ PULSED-AF reported 1 year effectiveness of 66.2 and 55.1% for PAF and PersAF patients respectively.^20^ Few other PFA technologies have reported data for treatment of persistent AF patients.^20,21^ While these studies report primary outcomes through 12 months, the survival estimates at 6 months in the VOLT CE Mark Study are similar to or higher than the 6-month survival estimates published by comparable PFA studies.^16,20–22^ Additionally, the highly acceptable rate of chronic success observed in the VOLT CE Mark Study at 6 months post-index procedure in PersAF patients was achieved with a PVI-only approach. Continued follow-up will be necessary to confirm the current comparative results.

It is also important to mention the low rate of PV reconnection in patients undergoing re-ablations during follow-up. Another study on the durability of PVI following PFA-based ablation showed a durable PV isolation rate of 69% of veins, and only 42% of the patients undergoing redo ablations had all PVs isolated, whereas the rate of durably isolated PVs in VOLT CE Mark Study patients with clinically relevant recurrence requiring re-ablation at any time was 82.6%.^23^

### Catheter features

The balloon-in-basket PFA catheter design provides unique advantages, including uniform electrode distribution and enhanced wall contact. This represents a potential improvement over current PFA technologies, where spline overlap or non-uniform spacing can create gaps in ablation lesions, increasing the risk of PV reconnection. Notably, the PV durability observed in our early findings with the Volt™ PFA catheter after recurrent remapping compares favourably to results reported in a previous study.^23^

Another significant advantage of the novel balloon-in-basket catheter is its real-time tissue contact monitoring and electroanatomic mapping system integration. This real-time display of the PFA catheter within the mapping system allowed for precise assessment of its position relative to the PVs and antral anatomy, thereby facilitating procedural workflows beyond fluoroscopy alone.^11^ The immediate feedback of tissue contact has the potential to optimize energy delivery at the PV level while minimising collateral damage.

### Limitations

This study has several limitations that should be considered when interpreting the results. First, the study employed a single-arm, non-randomized design, which limits the ability to draw direct comparisons with other treatment modalities or control groups. Second, the study cohort included a relatively low proportion of patients with PersAF, which may limit the generalizability of the findings, particularly for this patient population. Future studies with larger PersAF populations are needed to better assess the treatment's safety and effectiveness in this patient group. Overall, a sample size of 150 patients is a relatively small study population and may limit the generalizability of the safety and effectiveness data reported. Moreover, the incidence of rare complications, such as oesophageal fistula and coronary artery spasm cannot be evaluated due to the limited number of patients. Additionally, these findings evaluate 6-month effectiveness and the need for extended follow-up data is acknowledged. With long-term follow-up ongoing, the comparison of these early chronic effectiveness results to established PAF and PersAF ablation technologies will be further validated. Finally, the absence of mandatory remapping during follow-up to assess the comprehensive rate of PVI durability independent of clinical recurrence is another limitation. Although the study did not mandate remapping, the high rate of PVI durability at clinically driven repeat ablation was notable.

### Conclusion

The VOLT CE Mark Study demonstrates that the VOLT™ PFA system has a low rate of primary safety adverse events (2.7%) with no observed pulsed field energy-related complications. It also shows encouraging acute and chronic effectiveness, as well as a high PVI durability at clinically driven repeat ablation procedures. Long-term follow-up and randomized controlled trials are needed to further evaluate this system and allow for comparisons to other ablation energy sources, as well as to other PFA catheters.

## Supplementary Material

euaf072_Supplementary_Data

## Data Availability

The data underlying this article will be shared on reasonable request to the corresponding author in accordance with prevailing Human Research Ethics Committee approvals.
