# Supplementary material for: Safety and effectiveness of the first balloon-in-basket pulsed field ablation system for the treatment of atrial fibrillation: VOLT CE Mark Study 6-month results
Source: Europace. 2025 Mar 31;27(4):euaf072. doi: 10.1093/europace/euaf072 (PMC12036658; doi:10.1093/europace/euaf072)
Supplement: euaf072_Supplementary_Data [file euaf072_supplementary_data.docx]

# SUPPLEMENTAL MATERIAL

Primary Safety Event Details

Cardiac Tamponade: The left atrium was accessed via a transseptal puncture with the Agilis NxT Steerable Introducer Dual Reach guided by Ensite anatomical mapping, LivePoint, and fluoroscopy. A complex anatomy of the pulmonary veins was noted following the insertion of the Volt™ PFA catheter, and the physician found it difficult to make sufficient contact with the atrial tissue and probing via the guidewire was also difficult. Following PVI, the routine TTE showed a pericardial effusion that quickly transitioned to a pericardial tamponade requiring drainage without surgery.

Vascular Access Complications/Major Bleeding Events: One vascular access complication required left common femoral artery and external iliac artery repair. This patient experienced no further complication. The other event was classified as a major bleeding event due to right common femoral bleeding post-procedure and a computed tomography (CT) angiogram showing a retroperitoneal hematoma on the right side. The patient received a blood transfusion and upon continuous improvement, was discharged 3 days post-procedure.

Device and/or procedure related cardiovascular and/or pulmonary adverse event that prolongs hospitalization for more than 48 hours: This patient presented with pneumonia 5 days post index procedure and antibiotic therapy was administered.

**Supplemental Table 1.** Pulmonary Vein Reconnection During Index Procedure 20-minute waiting period

|  | **Treated Veins**  **(N=578)** |  |
| --- | --- | --- |
| Reconnected pulmonary veins during 20-min waiting period (Veins) | 1.7% (10/578) |  |
| LSPV | 50.0% (5/10) |  |
| LIPV | 10.0% (1/10) |  |
| LCPV | 0.0% (0/10) |  |
| RSPV | 20.0% (2/10) |  |
| RIPV | 20.0% (2/10) |  |
| Other | 0.0% (0/10) |  |

**Supplemental Table S2.** Reason for 6-Month Effectiveness Endpoint Failure

|  | **PAF  (N=103)** | **PersAF  (N=43)** | **PFAD  (N=146)** |  |
| --- | --- | --- | --- | --- |
|  |  |  |  |  |
| **Documented AF/AFL/AT Recurrence** | 11.7% (12/103) | 23.3% (10/43) | 15.1% (22/146) |  |
| AF | 5.8% (6/103) | 23.3% (10/43) | 11.0% (16/146) |  |
| AFL | 1.0% (1/103) | 4.7% (2/43) | 2.1% (3/146) |  |
| AT | 4.9% (5/103) | 0.0% (0/43) | 3.4% (5/146) |  |
| **Use of a new class I/III AAD** | 0.0% (0/103) | 7.0% (3/43) | 2.1% (3/146) |  |
| **Use of a class I or III AAD at a dose higher than that previously failed** | 1.0% (1/103) | 4.7% (2/43) | 2.1% (3/146) |  |
| **Repeat procedure >80 days post index procedure** | 1.0% (1/103) | 4.7% (2/43) | 2.1% (3/146) |  |
| **Cardioversion for treatment of AF** | 1.0% (1/103) | 4.7% (2/43) | 2.1% (3/146) |  |
| **Failure to achieve acute procedural success** | 0.0% (0/103) | 4.7% (2/43) | 1.4% (2/146) |  |
| **Second repeat procedure ≤80 days post index procedure** | 0.0% (0/103) | 0.0% (0/43) | 0.0% (0/146) |  |
| **Any ablation in the left atrium using an ablation catheter other than Volt PFA Catheter** | 0.0% (0/103) | 0.0% (0/43) | 0.0% (0/146) |  |
| **Surgical intervention for pre-existing arrhythmia** | 0.0% (0/103) | 0.0% (0/43) | 0.0% (0/146) |  |
| **Total 6-Month Effectiveness Failures** | 13.6% (14/103) | 27.9% (12/43) | 17.8% (26/146) |  |
|  | | |  |  |

A subject could fail on multiple endpoint components on the same day; thus the categories are not mutually exclusive.

**Supplemental Table S3.** Repeat ablations

|  | **PAF (n=103)** | **PersAF (n=43)** | **All (n=146)** |  |
| --- | --- | --- | --- | --- |
|  |  |  |  |  |
| **Patients with repeat procedure for AF/AFL/AT recurrence** | 3.9% (4/103) | 7.0% (3/43) | 4.8% (7/146) |  |
| **Number of Repeat procedures for AF/AFL/AT recurrence** | 4 | 3 | 7 |  |
| **Number of procedures 31-80 days post index procedure** | 50.0% (2/4) | 33.3% (1/3) | 42.9% (3/7) |  |
| **Number of procedures >80 days post index procedure** | 50.0% (2/4) | 66.7% (2/3) | 57.1% (4/7) |  |
| **Average days since initial procedure**        Mean ± SD (*n*)        Range (Min, Max) | 131.0 ± 15.6 (2)  (120, 142) | 113.0 ± 1.4 (2)  (112, 114) | 122.0 ± 13.8 (4)  (112, 142) |  |
|  | | |  |  |

**Supplemental Table S4.** Reconnected veins during repeat ablations with left atrial remapping

|  | **PAF  (N=103) (V=410)** | **PersAF  (N=43) (V=168)** | **All  (N=146) (V=578)** |  |
| --- | --- | --- | --- | --- |
|  |  |  |  |  |
| Subjects with Repeat Procedure and Left Atrial Remapping Completed | 2.9% (3/103) | 7.0% (3/43) | 4.1% (6/146) |  |
| Originally Isolated PV's Ablated with Reconnections upon Remapping | 8.3% (1/12) | 27.3% (3/11) | 17.4% (4/23) |  |
| Location of Reconnected Pulmonary Veins    LSPV | 0.0% (0/3) | 50.0% (1/2) | 20.0% (1/5) |  |
| LIPV | 0.0% (0/3) | 50.0% (1/2) | 20.0% (1/5) |  |
| RSPV | 33.3% (1/3) | 0.0% (0/3) | 16.7% (1/6) |  |
| RIPV | 0.0% (0/3) | 33.3% (1/3) | 16.7% (1/6) |  |
| Left Common | 0.0% (0/0) | 0.0% (0/1) | 0.0% (0/1) |  |
| Other | 0.0% (0/0) | 0.0% (0/0) | 0.0% (0/0) |  |
|  | | | |  |

AAD-Free Effectiveness

AAD-free 6-month success is defined as the primary composite effectiveness endpoint except that any use of Class I or III AADs after the 90-day blanking period is also considered a failure. 75.6% of PAF subjects and 60.5% of PersAF subjects were considered an AAD-free 6-month success.

To further distinguish effectiveness of the index procedure ablation independent of AADs, freedom from recurrence was evaluated in patients who discontinued all AADs post-blanking period separately from those who were on AADs at any time during follow-up. In PAF and PersAF subjects who were completely off Class I/III AADs post-blanking period through the 6-month cutoff, 89.7% (78/87) and 84.4% (27/32) were free from AF/AFL/AT recurrence as determined by protocol-specified monitoring, respectively. In PAF and PersAF subjects who took Class I/III AADs at any point post blanking period through the 6-month cutoff, 81.3% (13/16) and 54.5% (6/11) were free from AF/AFL/AT recurrence as determined by protocol-specified monitoring, respectively.
